# Evaluating the economic and social impact of coal mine phaseout in China

**DOI:** 10.1016/j.fmre.2025.09.001

**Published:** 2025-09-11

**Authors:** Bin Zhang, Niu Niu, Qingyao Xin, Yang Lu, Heran Zheng, Zhaohua Wang

**Affiliations:** a School of Management, Beijing Institute of Technology, Beijing 100081, China; b The Bartlett School of Sustainable Construction, University College London, London WC1E7HB, UK; c School of Economics, Beijing Institute of Technology, Beijing 100081, China

**Keywords:** Coal mine phaseouts, Regional imbalance, Coal mine-level inventory, Economic cost, Health benefits

## Abstract

Systematic and widespread coal mine phaseouts are a crucial step in facilitating the transition from coal dependence in China. However, regional disparities in economic reliance on coal and environmental impacts exacerbate the uneven social costs of phaseouts. A fair and effective trajectory for coal mine phaseouts is needed, but remains unclear. This study develops an interdisciplinary framework, incorporating a coal mine-level inventory, to assess the economic costs, health benefits, and regional disparities of two phaseout strategies: the Efficiency Priority Strategy (EPS) and the Equitable Reduction Strategy (ERS). The EPS focuses on phasing out inefficient and heavily polluting coal mines, while the ERS distributes capacity reductions more evenly across all coal mines. The findings reveal a significant imbalance in the distribution of costs and benefits, particularly in core coal-producing regions, which are often economically underdeveloped. These regions bear a disproportionately high share of stranded asset costs while receiving comparatively lower health benefits. Under the ERS, stranded asset costs in core coal-producing regions rise by 25.8%, increasing their share of national costs from 67.9% to 92.1%. Meanwhile, health benefits dropped by 12.7%, reducing their national share from 10.6% to 9.8%. This study provides valuable insights for China and other coal-dependent nations to develop more equitable coal mine phaseout policies, striking a balance between economic costs and health benefits within carbon budget constraints.

## Graphical abstract

**Figure:**
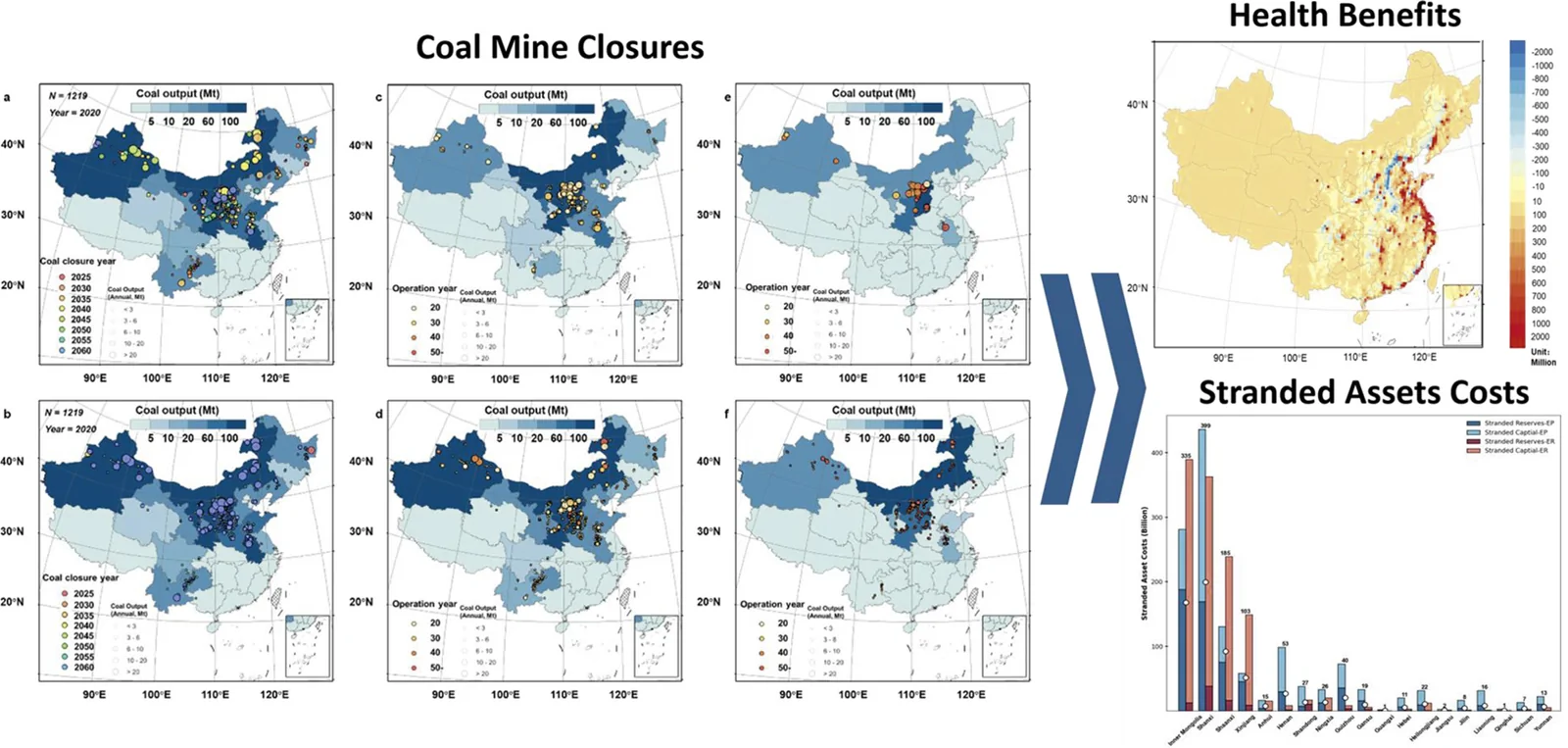

## 1 Introduction

Coal-related emissions account for approximately 40% of global greenhouse gas emissions [1], making the coal phaseout a critical step in mitigating climate change. By 2050, coal consumption must be reduced to just 18% of its 2020 levels [2], necessitating that 90% of existing coal reserves remain unexploited [3]. These ambitious targets introduce significant uncertainty for coal mining operations. As the world’s largest producer and consumer of coal, China faces immense pressure to reduce its coal demands. In response, China has pledged to achieve carbon neutrality by 2060 and peak carbon emissions before 2030. While ensuring energy security, the country is strengthening regulations on coal production, gradually reducing its dependency on coal, and systematically expanding alternative energy sources. Despite these commitments, the structural characteristics of China's coal sector pose challenges to a phased, orderly transition. Most operational coal mines have been in service for fewer than 15 years [4], well below their expected lifespans. This mismatch could create short-term oversupply, potentially leading to a lock-in effect that hinders the transition to sustainable energy alternatives [[5], [6], [7]]. In the long term, the imbalance between current coal supply and projected future demand introduces substantial economic risks, including stranded assets, underutilized infrastructure, and fiscal instability in coal-dependent provinces [7,8]. Research indicates that to align with the 1.5 °C climate target, China must leave over 60% of its coal reserves unmined by 2050 [3]. The anticipated stagnation and subsequent rapid decline in coal consumption imply that a significant portion of coal resources will remain unmined. This will lead to stranded assets and substantial value losses for entities holding these reserves [9].

Coal mining also impacts surrounding air quality [10,11]. In economically underdeveloped coal-producing regions of China, extensive coal mining activities substantially degrade air quality, releasing particulate matter that penetrates deep into the respiratory tract, causing severe respiratory and cardiovascular disorders [12]. Despite their economic reliance on coal, these regions endure disproportionately severe health risks due to sustained environmental degradation. Research indicates that halting coal mining could reduce local air pollution by as much as 8% [13]. In response, key coal-producing regions have introduced stricter air quality control measures to mitigate pollution associated with mining activities and curb its long-term environmental and health consequences. Additionally, the storage and transportation of coal contribute to respiratory diseases, increasing premature mortality rates by 1.1% and 1.3%, respectively [14,15]. The paradox of enduring severe pollution while remaining economically marginalized highlights a critical disparity. The effects of coal mining extend far beyond the immediate vicinity of the mines. Particulates can travel hundreds or even thousands of kilometers [16], degrading air quality and increasing health risks in distant regions [17]. While studies have explored the environmental and health benefits of regulating coal use in power generation [[18], [19], [20], [21], [22]], comprehensive research on reducing coal mining in China’s air quality and associated health benefits remains limited.

China’s extensive coal production and the entrenched dependency of its energy systems on coal amplify the uncertainties surrounding coal mine phaseouts. These uncertainties stem primarily from the inherent tension between equity and efficiency in the phaseout process [23,24]. On the one hand, the Efficiency Priority Strategy (EPS) focuses on phasing out high-cost, highly polluting mines, which could disproportionately burden economically vulnerable regions and widen regional development disparities. On the other hand, the Equitable Reduction Strategy (ERS) aims to distribute phaseouts more evenly across the country, but it risks allowing high-pollution, high-cost mines to continue operating, thereby undermining emissions reduction and public health goals. Moreover, China’s coal-dependent regions often have a narrow industrial base and lagging economic development. Poorly planned phaseout strategies risk exacerbating regional disparities, further entrenching economic inequalities.

Existing studies have examined coal mine phaseout strategies under the 1.5 °C and 2 °C climate targets, primarily focusing on the global distribution of production reductions and their alignment with climate commitments. These analyses often highlight the need to define coal production geographies within the framework of common but differentiated responsibilities among nations, emphasizing disparities in mitigation obligations between coal-producing and non-coal-producing regions [3,25,26]. While global-scale studies offer important insights into overall transition pathways, they do not fully capture the country-specific challenges of coal mine closures, especially in regions where coal production is concentrated and integral to local economies. At the national level, implementing coal mine phaseouts requires a more targeted approach that balances climate goals with economic stability and regional equity [27]. However, research on equitably and efficiently phasing out coal mines within individual countries remains limited. Most existing studies on equity and efficiency in coal phaseouts have focused on consumption, assessing the impacts of reducing coal use in power generation and industry. In contrast, the supply-side phaseout process—particularly at the coal mine level—has received comparatively less attention. Recent studies have utilized coal mine-level data to examine specific environmental impacts [28,29], such as methane emissions from active mines, and have identified opportunities for targeted reductions in emissions [28]. Additionally, while previous research has acknowledged the need to balance efficiency- and equity-based approaches to coal phaseouts, much of the discussion has remained conceptual, with limited empirical analysis or scenario-based policy evaluation. The development of robust, quantitative indicators to guide the prioritization of mine closures based on economic and social considerations remains an area requiring further investigation.

This study advances the literature by providing a coal mine-level quantitative assessment of the economic-health trade-offs associated with coal mine phaseouts, addressing critical gaps in existing research. Unlike previous studies focusing on macro-level production pathways or consumption-side policies, this study adopts a comprehensive supply-side perspective, systematically evaluating phaseout strategies across China’s coal mining sector. By examining the economic costs and health benefits associated with different phaseout strategies, this study offers a detailed analysis of how these approaches influence regional disparities, providing critical insights into the complex trade-offs involved in coal mine closures. First, we simulate the Global Change Assessment Model (GCAM) with a comprehensive dynamic coal mine database to design two coal mine phaseout strategies aligned with China’s carbon neutrality target: the EPS and the ERS. Subsequently, to quantify the regional health benefits under these strategies, we employed the Weather Research and Forecasting Model (WRF) coupled with the Comprehensive Air Quality Model with Extensions (CAMX) and the Global Exposure Mortality Model (GEMM). Our findings show that by 2060, stranded asset costs from coal mine phaseouts are estimated to be 123.7–133.3 billion yuan, potentially preventing 300,000–315,000 premature deaths and generating health benefits of 347.8–368.9 billion yuan. However, despite its goal of evenly distributing phaseouts, the ERS paradoxically intensifies regional inequities. Stranded asset costs in core coal-producing regions increase by 25.8%, while health benefits decline by 12.7%, further exacerbating the financial and health burdens of these disadvantaged regions. These results underscore the need for phaseout strategies that balance efficiency and equity, offering critical insights for policymakers in China and other coal-dependent nations seeking a just energy transition.

## 2 Method

We first constructed a coal mine-level database and developed an integrated assessment framework, as illustrated in Fig. 1. This framework supports the formulation of pathways toward carbon neutrality. It facilitates the evaluation of two distinct coal mine phaseout strategies, assessed through efficiency and equity. Detailed descriptions nof these methodologies are provided in subsequent sections and supplementary materials.

**Fig. 1:**
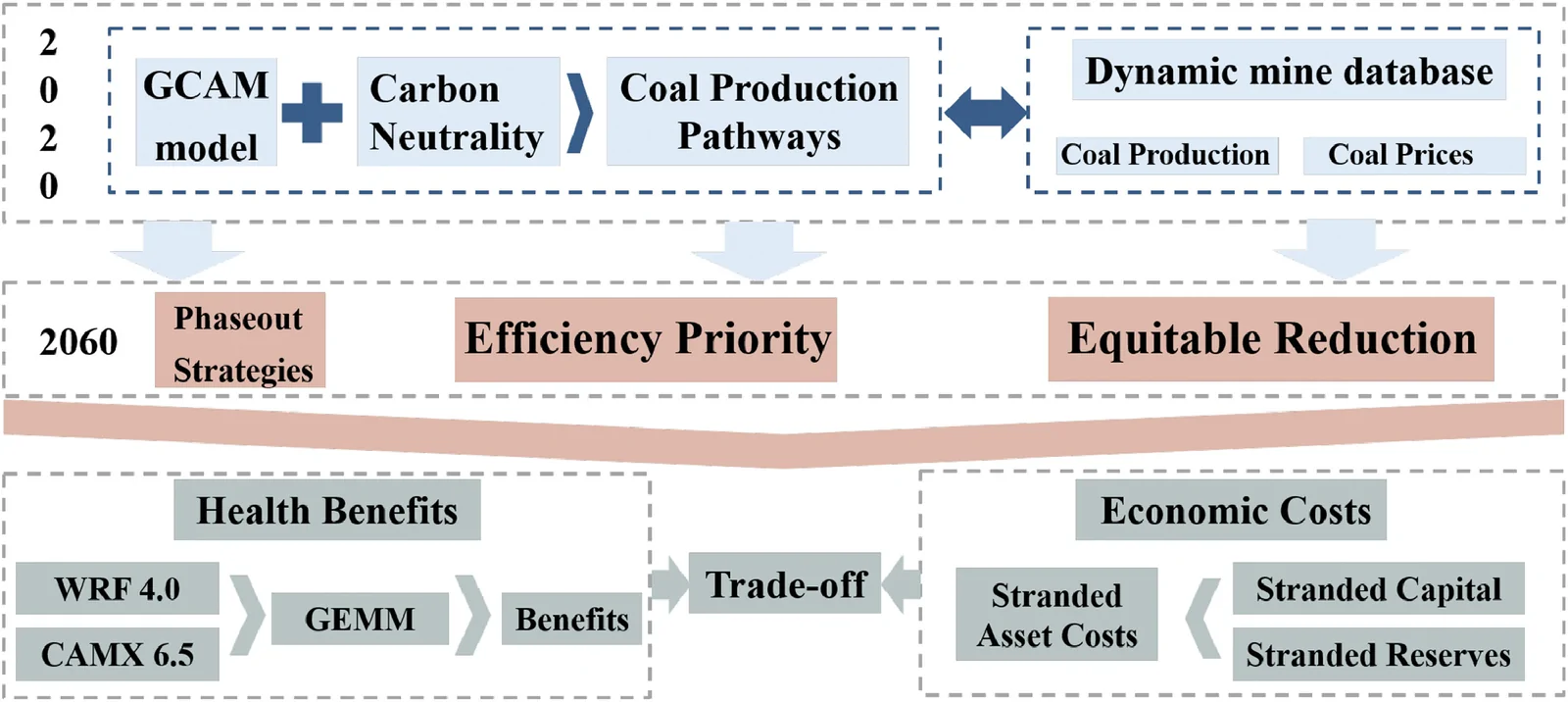
**Integrated assessment framework of this study.** First, the GCAM model is employed to simulate coal mine production pathways under the carbon neutrality scenario, which, in conjunction with the Dynamic Mine Database, is used to develop two distinct phaseout strategies. The WRF4.0-CAMX6.5 modelling system is then applied to quantify air pollution changes under each phaseout strategy. Subsequently, the GEMM model is utilized to estimate the health benefits associated with these changes in air quality. Additionally, the stranded capital and stranded reserves of coal mines are calculated for each phaseout strategy. Finally, the health benefits and economic costs under different phaseout strategies are derived, providing a comprehensive assessment of their implications.

### 2.1 Scenario development

We utilize the GCAMV6.0 to develop a carbon neutrality scenario for China, quantifying coal production under carbon neutrality [30]. GCAM, a sophisticated global integrated assessment model, simulates interactions among energy systems, water resources, agriculture, land use, economies, and climate dynamics to project future trajectories. This model has been widely employed in climate scenario studies and serves as one of the cornerstone models in Intergovernmental Panel on Climate Change (IPCC) assessments, underpinning the Representative Concentration Pathways [31] (RCP) and Shared Socioeconomic Pathways [32] (SSP) frameworks. Following the net-zero emissions trajectory outlined for 2060 by Fuhrman et al. [33]., this study models a linear reduction in China’s CO_2_ emissions from 2020, aiming to achieve net-zero status by 2060. This methodological approach is extensively validated and widely recognized in current scholarly research [34,35]. Details of the scenario ensembles are provided in Supplementary Note S2**.**

### 2.2 Coal mine phaseout strategy development

In assessing individual coal mines, we employ a multi-criteria decision-making method that incorporates factors such as operational duration, annual output, extraction methods, and coal grade. Detailed scoring criteria for each dimension are provided in Supplementary Note S3.

We developed two distinct coal mine phaseout strategies based on the principles of efficiency and equity. The principle of efficiency involves prioritizing the closure of mines based on performance metrics related to cost and pollution intensity. The principle of equity involves distributing required production cuts more broadly across regions or mines. Based on these considerations, the two strategies are defined as follows:

**Efficiency priority strategy:** Under this strategy, coal mines are ranked based on performance scores (For further details, please refer to Supplementary Note S3). Phaseout proceeds sequentially, starting with the lowest-scoring mines, which are identified as the most inefficient and polluting, until the required cumulative production reduction under the carbon neutrality pathway is met for each period.

**Equitable reduction strategy:** In contrast to the efficiency focus of EPS, the ERS represents a scenario designed to explore the implications of a burden-sharing approach based primarily on production scale. This strategy incorporates a key directive from China’s relevant national policy on coal mine disposal [36], mandating the closure of mines with an annual capacity below 0.3 million tons. The referenced policy includes mandatory closures based on various criteria and policy-guided voluntary exits, as well as potential mine upgrades or retrofits under specific conditions. Explicitly modelling these complex, mine-specific options, however, exceeds the scope of our large-scale comparative assessment framework, which focuses on contrasting broad strategic principles. Consequently, the ERS simulated herein adopts a necessary simplification. Following the mandated closures of mines with a capacity below 0.3 million tons, the remaining required national production decrease is allocated proportionally based on production capacity across all other operational mines.

### 2.3 Coal mine-based emission accountings

We quantify the emissions of key pollutants, including PM_2.5_, NO_X_, SO_2_, PM_10_, and PMC, of coal mines by correlating their annual production metrics with specific emission factors (Supplementary Note S4). The emissions for each pollutant type from coal mine i in 2020 are calculated using the formula:

(1) $$E_{m,t}=P_{m}\times F_{m,t}$$

where $E_{i,t}$ denotes the pollutant temissions of coal minem, $P_{m}$represents the annual output of coal minem, and$F_{m,t}$ signifies the corresponding emission factor for pollutant tof minem.The emission factors are derived from a rigorous review of existing literature.

We integrate carbon neutrality scenarios with WRF and CAMX to simulate surface PM_2.5_ concentrations. This integration, implemented through the WRF-CAMX framework, ensures high precision in modelling PM_2.5_ dynamics across China. Simulations are conducted over a 36 km × 36 km grid, encompassing all of China and its neighboring regions, with 30 vertical layers. Annual mean levels are derived by averaging hourly simulation outputs for four representative months: January, April, July, and October. WRF4.0 was used to provide meteorological fields for CAMX simulations in 2020. Emission inputs for China are sourced from MEIC, while emissions from neighboring countries are obtained from the Mosaic Asian Anthropogenic Emission Inventory. Natural emissions are derived from the Model of Emissions of Gases and Aerosols from Nature V2.1, while emissions from open biomass burning are taken from the Fire Inventory from the NCAR database. Boundary chemical conditions are initialized using the Whole Atmosphere Community Climate Model. Detailed information on model parameters, meteorological configurations, and chemical mechanisms is provided in Supplementary Note S5.

### 2.4 PM _2.5_ associated human health impacts evaluation

We then quantify the health impacts attributable to PM_2.5_ exposure. Baseline population data for 2020 and projected grid-based population data for China under SSP1 are derived from the Global Grid Population Database, featuring a high-resolution spatial grid of 1 km x 1 km. Age group segmentation is informed by the data from the Seventh Census [37], ensuring demographic precision in our assessments. To enhance the accuracy of outdoor PM_2.5_ exposure simulations, we employ the GEMM, as proposed by Burnett et al. [38]., which facilitates robust estimation of relative risks (RR) associated with particulate matter exposure (See Supplementary Note S6).

(2) $$RR\left(C\right)=e^{\frac{\theta \times \ln\left(\frac{z}{\alpha }+1\right)}{1+e\left(-\frac{z-\mu }{\nu }\right)}},Z=\max\left(0,C-2.4\right)$$

where C represents the ambient PM_2.5_ concentration,θ,α,μ,ν are used to define the shape parameters for the RR within the GEMM for different age groups, edenotes the natural logarithm. In alignment with recent scholarly findings, the theoretical baseline for minimum risk exposure to PM_2.5_ employed in our study corresponds to 2.4 µg/m³. Parameters furnished by GEMM dictate that the relative risk for non-communicable diseases (NCD) and lower respiratory infections (LCI) applies to individuals aged 25–85, assessed in five-year increments, whereas chronic obstructive pulmonary disease (COPD), lung cancer (LC) and stroke are the mean values for ages 25–85 are adopted. The estimated mortality rates for population groups within grid i categorized by gender g and age a are computed as follows:

(3) $$Mort_{i,j,g,k}=\sum_{d}\frac{RR{\left(P\right)}_{i,y,j,d}-1}{RR{\left(P\right)}_{i,y,j,d}}\times POP_{i,j,g}\times MB_{i,j,g,k}$$

where $Mort_{i,j,g,k}$ represents the number of deaths in grid i for age group j, gender g,and disease k; $POP_{i,j,g}$denotes the population count for age group j and genderg in grid i; $MB_{i,j,g,k}$ indicates the morbidity rate for disease k in age group j and gender g within grid i. We establish baseline mortality rates for five primary causes of death using the GBD 2019 database [39]: COPD, IHD, LC, LRI and Stroke (See Supplementary Note S6 Table S7 for the historical and future age-specific baseline mortality.).

Ultimately, to quantify the health benefits accruing from reductions in PM_2.5_ exposure, we utilize the Value of Statistical Life (VSL) (See Supplementary Note S6, Table S8 for provincial VSL.). This methodology facilitates the economic valuation of health outcomes, thereby translating the epidemiological reductions in morbidity and mortality into financial terms.

(4) $$VSL_{p,2060}=VSL_{p,2020}\times {\left(\frac{PerGDP_{p,2060}}{PerGDP_{p,2020}}\right)}^{elasticity}$$

where $VSL_{p,2060}$ represents the Value of Statistical Life for province p in 2060, while $VSL_{p,2020}$ denotes the Value of Statistical Life for province p in 2060. Drawing from the research by Jiang et al. [40]. $PerGDP_{p.2020}$ signifies the per capita GDP of province p in 2020, and $PerGDP_{p.2060}$ indicates the per capita GDP of province p in 2060. elasticity refers to the elasticity coefficient of VSL relative to per capita GDP, for which we have selected a value of 0.8 [41].

### 2.5 Stranded asset costs

The economic implications of stranded coal mine assets are delineated into two principal categories: stranded coal reserves and stranded capital [9]. To quantify the stranded fossil fuel reserves, the discounted cash flow model is employed. This approach facilitates the valuation of specified quantities of reserves by calculating the present value of projected cumulative revenues from the exploitation of fossil fuels.

(5) $$FR_{m}=\sum_{y=0}^{Y}\frac{\left(C_{y}-MPC_{y}\right)\times TR_{m}\times O_{m,y}\times \frac{RY_{m}}{SC_{m}}}{{\left(1+d\right)}^{y}}$$

where $FR_{m}$ denotes the cost associated with stranded reserves, $c_{y}$ represents the market price of coal in yyear, $MPC_{t}$ stands for the marginal cost of extraction in y year, $TR_{m}$ indicates the proven reserves of coal mine m, and $O_{m,y}$ signifies the proportion of unmined coal in year y relative to the proven reserves of coal mine m. The discount rate (d) is set at 10%. $RY_{m}\;$ represents the operational duration of the coal mine m, $SC_{m}$ signifies the service life of the mine m. For specific parameter selections, please refer to Supplementary Note S7.

We assume that the coal mine will receive no further investment, viewing stranded capital costs as the financial losses associated with unutilized capital assets. We measure the stranded capital costs by the discrepancy between the original cost of fixed assets and their net residual value.

(6) $$SA_{m}=\frac{FA_{m}}{{\left(1+d\right)}^{t}}$$

where $SA_{m}$ denotes the stranded capital costs, $FA_{m}$ denotes the present value of the fixed assets for coal mine m.

### 2.6 Sensitivity analysis

We conducted a sensitivity analysis to evaluate the robustness of our main findings to uncertainties in key model assumptions. This analysis addresses three primary sources of uncertainty. First, our results are sensitive to the assumed long-term socio-economic development pathway, which determines key inputs such as population growth and GDP per capita. These, in turn, influence the estimation of the VSL. To address uncertainty in benefit monetization, we introduce an alternative high-VSL scenario based on established national and international estimates [42], representing the upper bound of plausible valuation ranges. Second, future coal prices remain highly uncertain under the carbon neutrality target due to the dynamic interactions between global markets, domestic energy policies, and technological substitution [43,44]. Coal prices directly affect the economic valuation of stranded coal reserves. We incorporate alternative coal price trajectories, representing high-price and low-price scenarios, into the sensitivity analysis to explore this dimension. Third, the discount rate used to calculate the present value of future costs is a key parameter that is highly controversial in valuation practice [45]. To explore this, our sensitivity analysis examines the impact of different discount rates. Detailed descriptions of the specific parameter ranges tested, along with the methodology and results for each sensitivity analyses, are provided in Supplementary Note S8.

## 3 Results

### 3.1 Coal mine phaseout strategies and regional imbalance

The spatial distribution, annual production capacities, and operational durations of 1219 coal mines across China in 2020 reveal significant unevenness, with a pronounced concentration in economically underdeveloped regions (see Supplementary Note S1, Fig. S1 and S3). An inverse relationship is evident between the distribution of coal mines and regional economic development. Mines with lower production capacities predominantly reside in Shanxi, Shandong, and Guizhou. Large-scale mining operations, however, are concentrated in less economically developed regions such as Shanxi, Inner Mongolia, Shaanxi, and Xinjiang (see Supplementary Fig. S1). By 2060, coal production is projected to decline from 75.7 EJ to 7.5 EJ under carbon neutrality scenario (see Supplementary Fig. S2). Under the EPS, the pace of coal mine phaseouts accelerates, with over 70% of mines expected to be decommissioned by 2040, reducing the total number to 369, primarily in core coal-producing provinces. By 2060, only 34 mines remain, 31 in core regions, contributing 92.4% of national coal production (see Fig. 2a, 2c, 2e). In contrast, the ERS results in a slower rate of phaseouts, with only 26 coal mines expected to be decommissioned by 2040, leaving 1193 mines still operational. By 2060, the total number of coal mines under ERS will decrease to 426, with core regions contributing 64.1% of the national coal output (see Fig. 2b, 2d, 2f).

**Fig. 2:**
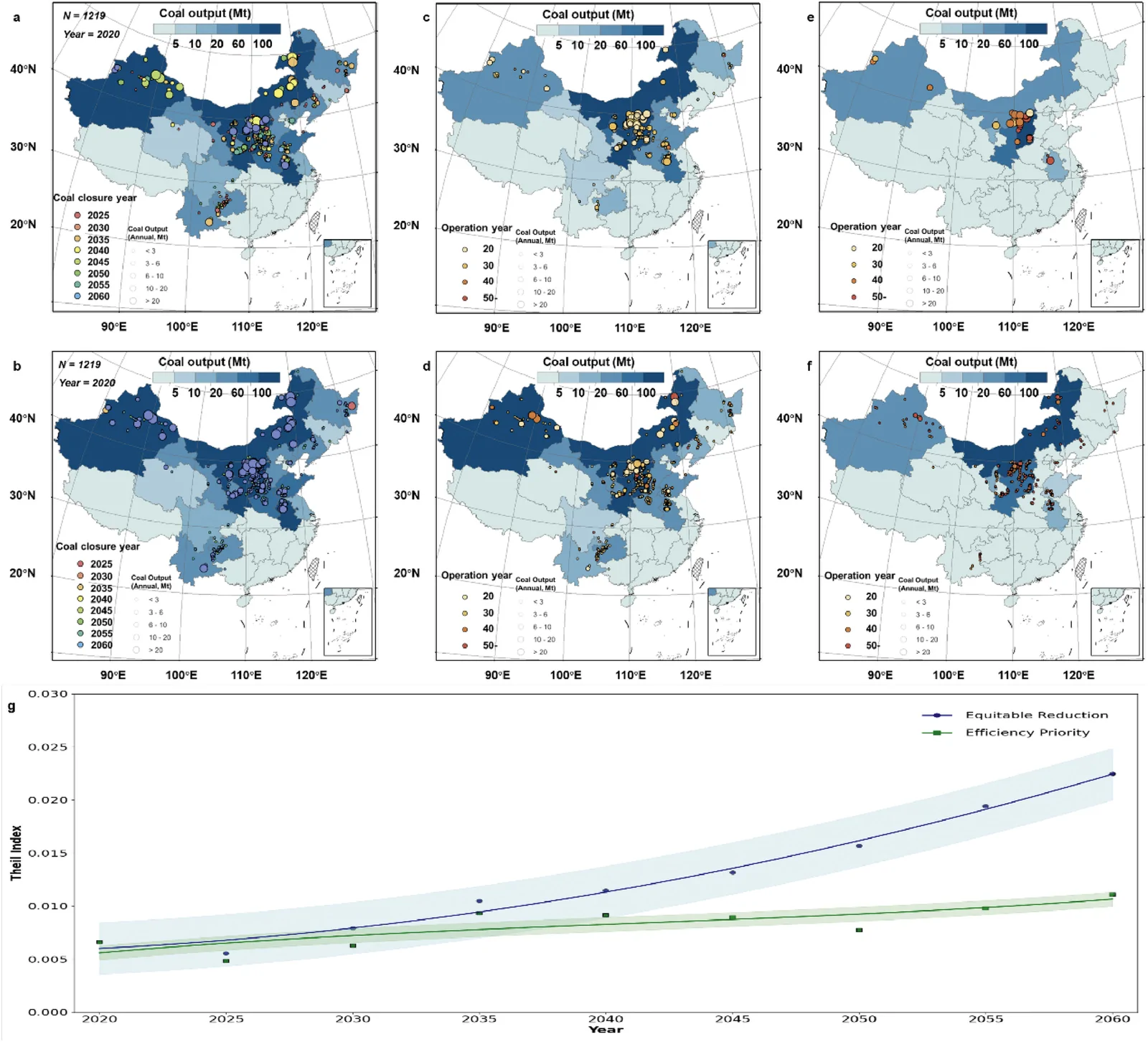
**Geographical distribution of coal mines in China in 2020 and the distribution of different coal mine phaseout strategies (GS(2024)0650).** (a) The geographical distribution and annual production of coal mines in China as of 2020, along with the phaseout year under the EPS. The size of each dot represents the mine’s annual production, while the dot color indicates the projected phaseout time. The background color shows the annual coal output of each province. The crosshatching indicates a lack of relevant data. The same applies below. (b) The geographical distribution and annual production of coal mines in China as of 2020, along with the phaseout year under the ERS. The size of each dot represents the mine’s annual production, and the dot color indicates the projected phaseout time. The background color shows the annual coal output of each province. (c–f) Coal production, distribution, and provincial coal outputs under the EPS and ERS for 2040 and 2060. (g) Theil’s index indicates the imbalance between coal production and economic development, with shading representing the 95% confidence band of the prediction using the fitted curve.

As coal mines are progressively phased out, the observed economic impacts on less developed coal-producing regions are more significant under either strategy than those on more economically developed areas. The increase in the Theil index indicates that the distribution of coal mining activities contributes to economic disparities in less developed regions during the transition, widening the gap with more developed regions (see Fig 2g). In contrast, many coal mines remain operational under the ERS beyond 2040. By that year, only 26 mines will have been phased out, leaving 1193 still operational (see Fig. 2d). The operational mines are mostly small in production capacity, and many have been in operation for over 50 years. A widespread phaseout of coal mines under ERS will occur after 2050, and by 2060, the total number will decrease to 426. The distribution of these remaining coal mines across China remains highly dispersed, with 16 provinces still maintaining operational mines. Only three provinces have entirely ceased coal operations compared to 2020 (see Fig. 2f). The core coal-producing areas contribute 64.1% of the total coal output under the ERS, a decrease of 28.3% compared to the EPS. This broader distribution under the ERS resulted in a more gradual reduction trajectory for many individual mines and a wider geographic spread of remaining coal production compared to the EPS.

### 3.2 Stranded asset cost and health benefit of coal mine phaseouts

Our analysis demonstrates a significant impact of coal mine phaseout on PM_2.5_ emissions, resulting in up to 0.7 µg/m³ reductions by 2060 (see Supplementary Fig. S4). However, these reductions exhibit substantial regional variations. In core coal-producing areas, the impact of mine phaseout on regional PM_2.5_ concentrations is relatively modest, ranging from 0.1 to 0.2 µg/m³. This modest improvement contrasts with more significant decreases of up to 0.7 µg/m³ in regions less synonymous with coal production, such as Hunan and Guangxi. This difference arises from several factors. In core coal-producing regions, persistent low wind speeds and temperature inversions hinder air circulation, thereby limiting the dispersal of pollutants. Additionally, low humidity in these areas reduces the removal of particulate matter through hygroscopic growth, further constraining PM_2.5_ reduction. PM_2.5_ concentrations remain consistently high in core coal-producing regions. Intense heavy industrial activity in these areas continuously emits significant pollutants, creating high-intensity pollution sources. Long-term high emissions have accumulated pollutants, limiting the effectiveness of new emission reduction measures in lowering PM_2.5_ levels. Under the EPS, national health benefits from coal mine phaseouts amount to 3689 billion yuan, exceeding the 3478 billion yuan achieved under the ERS by 6.1% (see Supplementary Fig S6). As illustrated in Fig. 2a, >70% of provinces (22) experience greater health benefits under the EPS. The EPS significantly enhances local health outcomes by prioritizing the phaseout of inefficient and heavily polluting mines in regions such as Shanxi. Economically developed regions such as Jiangsu and Guangdong also benefit from health improvements under the EPS, exceeding those under the ERS by 9.2% and 27.3%, respectively. However, regions like Beijing and Hebei, located downwind from core coal-producing provinces such as Shanxi and Inner Mongolia, experience diminished environmental health benefits under the EPS. By 2060, the concentration of coal mines in core regions under the EPS will transport pollutants via prevailing winds, reducing air quality improvements in downwind regions. While the ERS’s broader dispersion of mine phaseouts helps mitigate long-distance pollution transport, easing impacts in downwind areas, its widespread distribution of operational mines disproportionately impedes air quality enhancements in adjacent regions (see Supplementary Note S6 for aggregated health benefits by province). However, the health benefits from coal mine phaseouts within the province are significantly lower compared to the economically developed eastern regions. Population density and economic development markedly influence the geographic distribution of health benefits from PM_2.5_ reductions, revealing a significant mismatch between areas with substantial PM_2.5_ declines and those achieving notable health improvements. Densely populated and economically advanced eastern regions, such as Jiangsu (452–494 billion yuan) and Shanghai (260–261 billion yuan), reap the most benefits. In contrast, core coal-producing provinces like Shaanxi (248–265 billion yuan) and Inner Mongolia (68–75 billion yuan) gain less. >70% of provinces (22 in total) experience more significant health benefits under the EPS, which proactively closes less efficient and more polluting mines in regions like Shanxi, markedly boosting local health benefits.

Stranded asset costs are categorized into stranded coal reserves and stranded capital, as illustrated in Fig 3b The EPS maintains a relative balance between stranded capital (713.0 billion yuan) and reserves (620.5 billion yuan). In stark contrast, the ERS leads to disproportionately high stranded capital (1141.6 billion yuan)—over twelve times the value of its stranded reserves (95.3 billion yuan). This structural difference suggests that the ERS props up high-cost, low-yield operations for a longer period, indicating suboptimal resource allocation and potentially increasing financial vulnerability in dependent regions. Interestingly, while the ERS results in slightly lower (7.2%) total national stranded costs compared to EPS, this aggregate figure masks a severe concentration of the economic burden. Under ERS, core regions see their stranded asset costs increase by 7.2% (reaching 1139.4 billion yuan compared to 905.8 billion yuan under EPS), causing their share of the national total to increase dramatically from 67.9% to 92.1%. Inner Mongolia and Shaanxi, for instance, face the largest absolute cost increases under ERS, alongside significant rises in Xinjiang. This finding underscores how ERS can perversely deepen regional economic disparities during the phaseout. Consequently, considering both the distribution of these substantial costs and the previously discussed uneven distribution of health benefits, the two strategies engender distinct patterns of net economic loss risk across provinces. The EPS pathway concentrates on higher net loss risks, primarily in the provinces of Gansu, Guizhou, Ningxia, and Shanxi. Conversely, the ERS pathway shifts the most significant net economic loss risks towards Inner Mongolia and Xinjiang. This redistribution of economic vulnerability further highlights how fundamentally different phaseout principles translate into divergent and complex regional consequences.

**Fig. 3:**
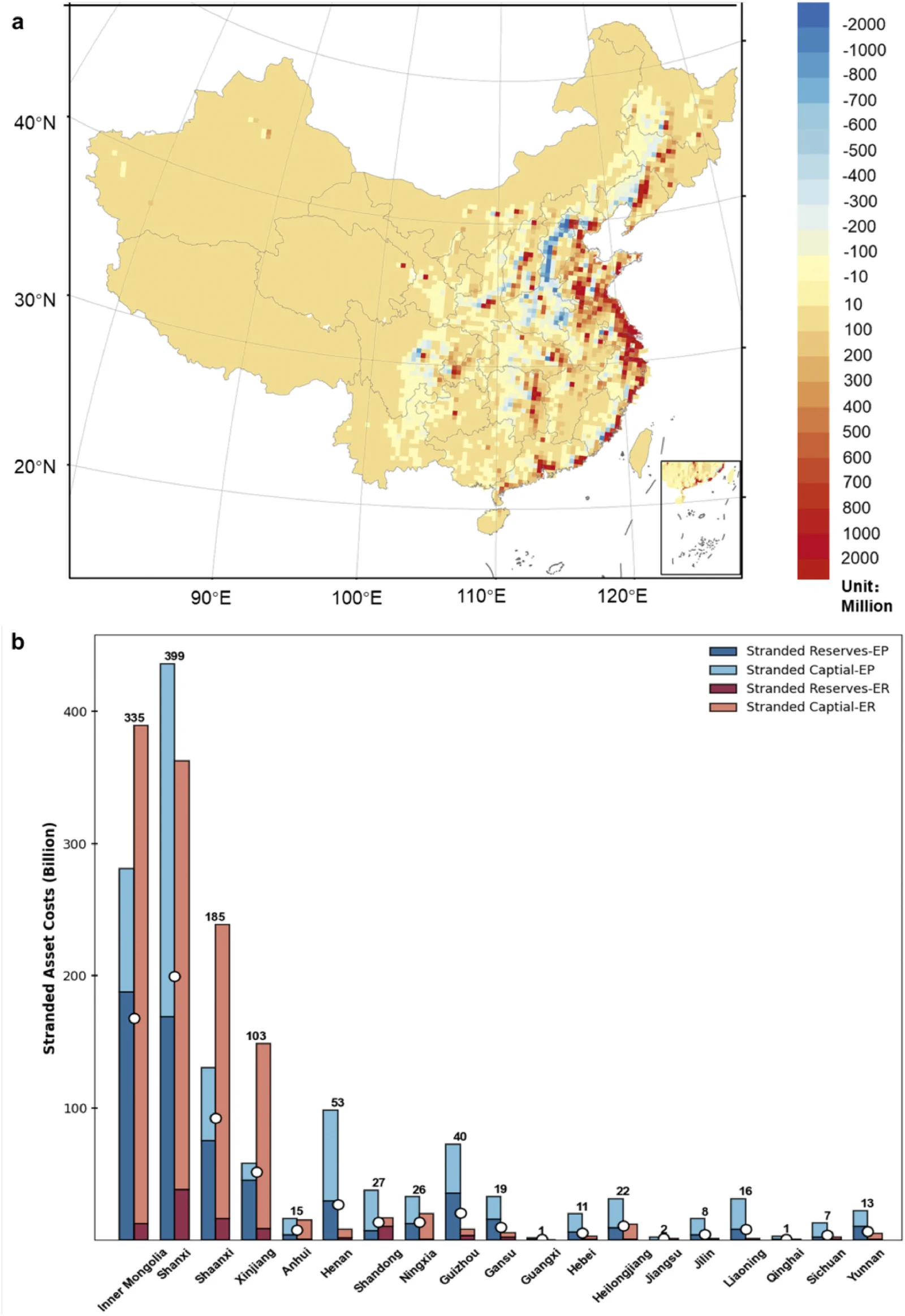
**Changes in PM_2.5_ concentration associated with coal mine phaseouts and changes in health benefit and stranded asset costs.** (a) The difference in health benefits from PM_2.5_ reductions following coal mine phaseouts between the EPS and ERS strategies. The colors represent regional differences in health benefits, with deeper red indicating greater benefits under the EPS compared to the ERS and deeper blue indicating greater benefits under the ERS (GS(2024)0650). (b) Stranded asset costs resulting from PM_2.5_ reductions following coal mine phaseouts under the EPS and ERS. Shades of blue represent the EPS, with dark blue corresponding to stranded reserve costs and light blue to stranded capital costs. Shades of red represent the ERS, with dark red corresponding to stranded reserves costs and light red to stranded capital costs. Dots in the diagram indicate the average stranded asset costs for each strategy.

To test the robustness of our findings, we conducted a sensitivity analysis to examine how variations in key parameters affect stranded asset costs and health benefits (see Supplementary Note S8). The results show that the discount rate affects stranded capital. Under the EPS, stranded capital is estimated to range from 596.14 billion yuan to 872.52 billion yuan, while under the ERS, it ranges from 783.30 billion yuan to 1775.33 billion yuan. Coal price fluctuations have an effect under the ERS, with stranded assets ranging from 9.35 billion yuan to 11.40 billion yuan, compared to 0.55 billion yuan to 0.74 billion yuan under the EPS. Adjustments to the VSL lead to changes in the national health benefits, with the EPS ranging from 3688.85 billion yuan to 5106.49 billion yuan and the ERS ranging from 3478.40 billion yuan to 4809.31 billion yuan (see Supplementary Figs. S6–S9).

### 3.3 The trade-off between health benefits and stranded asset costs

By 2060, health benefits from both coal mine phaseout strategies will sufficiently offset the associated stranded asset costs. The EPS delivers net benefits of 2355.4 billion yuan, 5.1% higher than the 2241.5 billion yuan yielded by the ERS. As shown in Fig. 4c and 4d, under the ERS, four provinces incur negative net benefits: Shanxi (−339.3 billion yuan), Inner Mongolia (−322.0 billion yuan), Xinjiang (−146.8 billion yuan), and Ningxia (−16.6 billion yuan). In contrast, non-coal-producing regions achieve net benefits of 1334.3 billion-yuan, accounting for 59.3%. While increasing the number of provinces with net losses, the EPS significantly alleviates financial burdens in core coal-producing regions, reducing total losses from 799.2 billion yuan to 516.2 billion yuan. The cost-benefit ratio across provinces also improves, with Shaanxi’ s ratio notably rising from 1.0 to 2.0. Additionally, non-coal-producing areas see a 7.3% increase in net benefits, rising to 1431.3 billion yuan.

**Fig.4:**
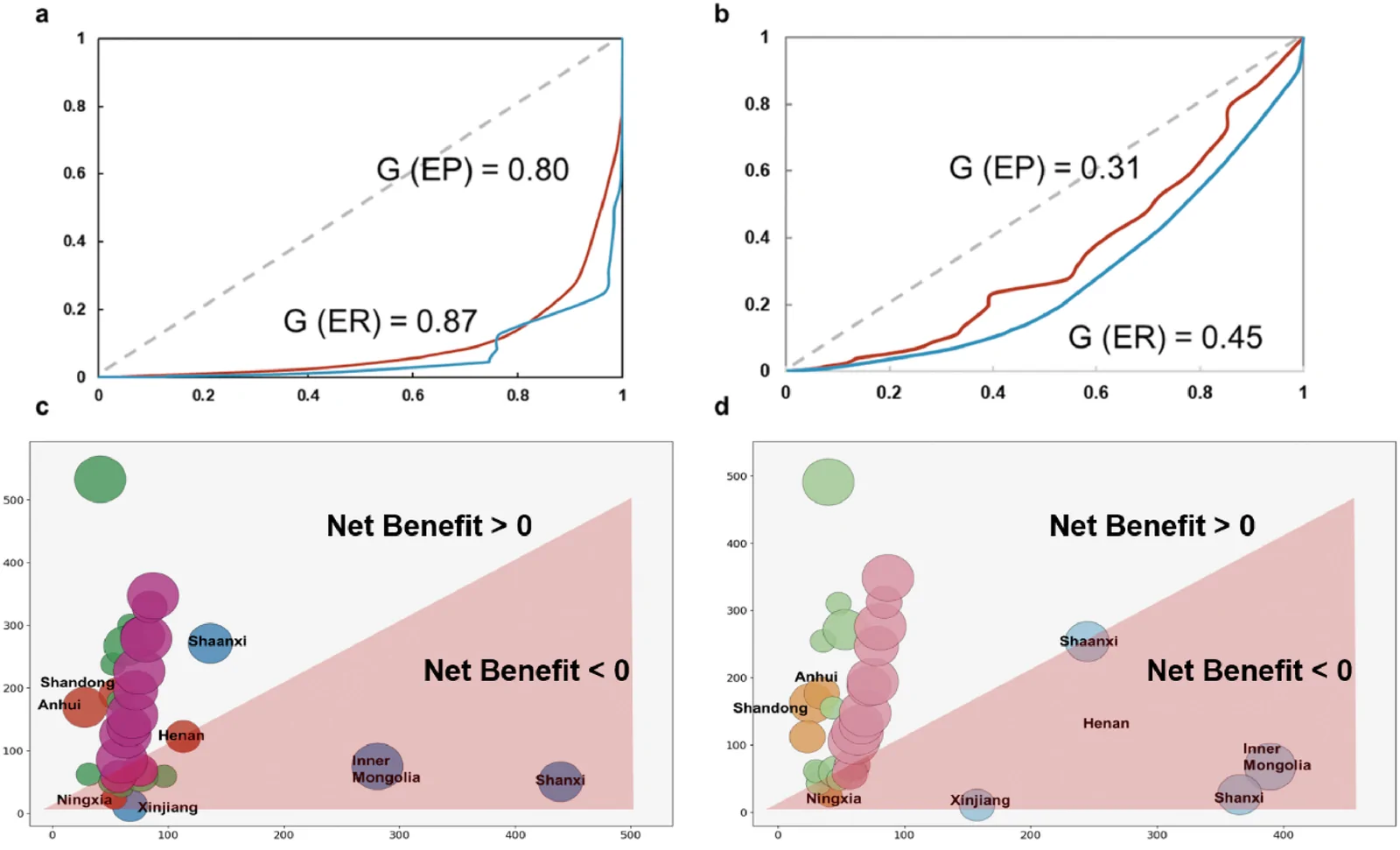
**Stranded asset costs and health benefits of coal mine phaseouts.** (a) Lorenz curve and Gini coefficient of coal mine phaseouts, showing the regional distribution of health benefits as a proportion of GDP. The red line represents the EPS, and the blue line represents the ERS. (b) Lorenz curve and Gini coefficient of coal mine phaseouts, showing the regional distribution of stranded asset costs as a proportion of health benefits in coal-producing regions. (c-d) Stranded asset costs and health benefits by province under the EPS and ERS. Shaded regions indicate where stranded asset costs exceed health benefits. Dark blue represents core coal-producing regions, light blue represents major coal-producing regions, red represents general coal-producing regions, and yellow represents regions without coal production.

We use the Gini coefficient (Fig.4, Fig.4) to analyze regional disparities during coal mine decommissioning. The total benefit Gini coefficients are 0.80 for the EPS and 0.87 for the ERS, respectively. This metric indicates that economically developed regions derive more substantial health benefits under the ERS. In coal-producing regions, the Gini coefficient under the EPS is lower at 0.31 compared to 0.45 under the ERS. This difference shows the EPS’s effectiveness in targeting inefficient mines for phaseout, which alleviates financial strains in coal-producing regions and improves the distribution of health benefits. By redistributing decommissioning pressures more evenly across provinces, the EPS supports the dual goals of economic sustainability and public health improvement, fostering more balanced regional development during the transition to carbon neutrality.

## 4 Discussion and policy implications

Climate equity is a cornerstone of climate policy formulation [23,46]. However, translating this principle into effective phaseout strategies for vast and diverse coal sectors, such as China’s, remains a challenge. The ERS paradoxically exacerbated regional disparities in economic costs and health benefit distribution compared to the EPS. This adverse outcome underscores the limitations of employing simplified, non-selective equity metrics that fail to account for fundamental regional heterogeneities, particularly the distinct economic structures that differentiate resource-dependent regions heavily reliant on coal from more diversified economies. Specifically, the nuances of national guidelines [36] regarding “classified disposal,” such as mine upgrades or retrofits based on local conditions, are not fully reflected. The ERS places heavy burdens on vulnerable coal regions while potentially preserving inefficient operations, highlighting how simplistic rules can undermine equity. This calls for more nuanced strategies, as even efficiency-driven policies like EPS have complex distributional effects. Beyond the efficiency and equity principles, coal mine decommissioning decisions in China are also influenced by other important factors, adding complexity to phaseout pathways. Ecological protection requirements often necessitate closing mines within designated protected areas, even considering their economic performance [47]. Mine safety regulations are equally critical. Coal mines with poor safety records or high geological risks are often prioritized for early closure to prevent accidents, taking precedence over purely economic considerations [48]. Complementing these complex operational and environmental considerations, the profound social consequences of the phaseout demand commensurate attention, particularly the potential for large-scale miner unemployment [49]. A rapid phaseout of coal mining risks large-scale unemployment among miners, particularly among ageing workers with low skill transferability and limited alternative employment opportunities. These shocks often cascade beyond individual livelihoods, threatening the stability of coal-dependent communities throughout migration, declining tax bases, and the erosion of public services. The ripple effects of coal phaseout policies reveal employment losses in mining and downstream sectors, such as construction, chemicals, and coal-fired power generation [49]. Therefore, actively managing worker displacement and supporting affected communities through dedicated “Just Transition” policies is essential to achieving a feasible and socially sustainable energy transition pathway in China.

For coal-producing regions, particularly those characterized as resource-dependent, adjusting supply-side policies is crucial for achieving environmental goals and mitigating the social impacts associated with the coal phaseout. Robust social protection mechanisms should accompany the phaseout of subsidies for high-pollution, low-efficiency coal mines. Such an approach can facilitate the orderly exit of outdated operations while safeguarding vulnerable populations and preventing abrupt socio-economic collapse. Comprehensive financial support is critical, extending beyond stranded asset costs to include dedicated funding for worker support programs and community revitalization initiatives.

International experience provides useful references for these policy measures. In Germany’s Ruhr region, a combination of social dialogue, retraining initiatives, and a coal adjustment fund supported large-scale workforce transitions and regional economic diversification, significantly alleviating the negative social impacts of mine closures [50]. The authorities closely aligned job retraining and skills development programs with employment opportunities in new sectors. These sectors included environmental remediation, renewable energy, and advanced manufacturing. Similarly, in the United States, the POWER Initiative in the Appalachian region combined federal and local resources to fund retraining, entrepreneurship, and infrastructure investments. Between 2015 and 2020, this program helped over 6500 workers successfully transition to new jobs and supported the creation of >11,000 new positions, illustrating the importance of targeted investment and institutional collaboration for regional recovery [51].

For China’s coal-producing regions, it is equally important to offer accessible training and skills development opportunities that match emerging industries, including clean energy, modern agriculture, and digital sectors. Wage subsidies during training can further facilitate worker transition into quality employment. Such diversification efforts require foundational investments and targeted incentives to attract new industries, such as renewable energy manufacturing, battery storage, and carbon capture, thereby fostering alternative economic opportunities in areas previously dependent on coal. Encouraging active participation from local governments and communities in transition planning can improve policy relevance and public acceptance. In addition, stronger regional cooperation will be important for sharing knowledge on technological innovation and sustainable economic strategies, ultimately supporting a just and orderly transition.

In contrast to the focus on socio-economic restructuring and diversification crucial for resource-dependent regions, policies should focus on grid modernization and energy security for non-coal-producing regions as coal dependency declines. Upgrading long-distance transmission infrastructure would enhance the integration of renewable energy from transitioning coal regions, reducing reliance on coal-based electricity imports. Expanding energy storage capacity and implementing demand-side management would help stabilize the power supply and mitigate fluctuations associated with renewable energy sources. Accelerating renewable energy deployment and industrial electrification in these regions would support the national energy transition while ensuring stable economic growth.

A clear and phased exit strategy, developed in coordination with stakeholders, is necessary for coal enterprises. The focus should be on shutting down high-emission and low-efficiency mines first while allowing some transitional operations under stricter standards. In addition to meeting environmental targets, enterprises should actively participate in and contribute to managing the social transition, fulfilling their responsibilities to support affected workers and communities. This includes offering or co-funding relevant, certified retraining programs aligned with regional needs, contributing meaningfully to local employment services, and proactively engaging in the environmental remediation and responsible repurposing of former mining sites. Repurposing these sites for renewable energy storage can mitigate asset losses and support local economic adjustment. Companies should also work collaboratively with local governments, worker representatives, and communities to design site-specific transition plans that reflect local needs, thereby enhancing their social license to operate during the transition.

Recognizing the profound challenges coal-producing regions face in managing mine phaseouts is essential. Without a strategically planned decommissioning pathway, these regions risk incurring additional burdens in their pursuit of China’s carbon neutrality. A single industrial structure and low economic development levels characterize China’s coal-producing regions. Public awareness of health risks associated with environmental pollution in these regions is often limited, potentially skewing willingness-to-pay responses in surveys that fail to capture an understanding of long-term health consequences [52]. As a result, the VSL could be significantly overestimated in regions where the economy relies heavily on coal mining. Our evaluation considers the marginal costs of extraction when assets become stranded, assuming these costs remain unchanged. In practice, extraction costs will likely rise as mines exhaust the easiest deposits first and leave more difficult, costly reserves behind. Tighter environmental regulations and growing management expenses will further increase these costs, which may cause our estimates of stranded assets to be too low [53,54]. Rapid progress in carbon capture, storage, and renewable energy technologies continues to erode the economic viability of coal-fired power generation [55,56]. As these technologies advance, coal assets will likely become stranded even faster and on a larger scale than currently expected [57,58]. Policymakers need to adopt robust and flexible policy frameworks to manage these transitions.

Evaluating China’s coal phaseout requires recognizing its distinct national context within the global energy transition landscape. While sharing the imperative of decarbonization, China’s pathway diverges significantly from that of other major coal producers. Unlike the United States, where coal’s decline has been largely driven by market forces, notably shale gas competition and environmental regulations operating within a federated system often lacking unified federal planning for closures [59], or Australia, whose trajectory is heavily influenced by its status as a major coal exporter responding to fluctuating global markets and domestic renewable economics [60], China employs centralized, state-led strategic planning aimed explicitly at carbon neutrality. India has fossil energy endowments similar to those of China, but it still faces a unique challenge: balancing energy security with the growing demands of its economy and the pressures of climate change [61]. This fundamental governance difference renders centrally coordinated, criteria-based phaseout strategies, such as the EPS and ERS evaluated herein, particularly pertinent and feasible in China. Structurally, China’s extensive fleet of relatively young coal mines implies distinct stranded asset risks and economic adjustment costs compared to nations with older infrastructure, directly impacting the magnitude and distribution of economic impacts calculated in our study [62]. Furthermore, a limitation of the health impact assessment is its focus on benefits to the general population resulting from changes in regional ambient air quality. While relevant for national policy evaluation, this approach does not differentiate health impacts according to mine type, for instance, between surface and underground operations. It also fails to distinguish between distinct exposure pathways, specifically occupational exposure for miners, versus ambient exposure for residents. Due to limitations in emission factor data, we have not differentiated exposure pathways by mine type, which may lead to an overestimation of health benefits in regions with predominantly underground mines [63], such as Shaanxi. In these areas, the environmental benefits from PM_2.5_ reduction may be less significant. Moreover, the use of uniform SSP1 population projections and a single GDP growth scenario may obscure significant intra-provincial variations, such as disparities between remote mining towns and regional capitals. These variations, including factors such as the share of the workforce in the coal sector and the average age of miners, could impact the accuracy of VSL valuations and health risk estimates.

Therefore, future research should prioritize developing more comprehensive frameworks for designing and evaluating coal phaseout pathways, moving beyond the simplified equity approaches analyzed in this study. Essential components of such advanced frameworks include the integration of multi-dimensional criteria. Strategy assessment should extend beyond economic and environmental metrics to encompass distributional impacts, including effects on jobs, community well-being, and health equity, while accounting for variations in exposure pathways and coal mine conditions. It is equally important to consider the varying exposure pathways and coal mine conditions, which can differ significantly depending on the type of mining operations. Furthermore, to improve the accuracy of health impact assessments, future studies should utilize more precise emission factors that account for differences between coal mine types. To ensure that the analysis reflects real-world complexities, it is essential to integrate higher-resolution socioeconomic data, such as detailed demographic and economic data at the sub-provincial level. This would enable a more accurate understanding of regional disparities, including variations in workforce composition, age structure, and economic resilience. Finally, incorporating greater policy realism is also essential. For instance, quantitatively evaluating differentiated options, such as mine upgrades versus closures, based on national guidelines [36] and local contexts, is crucial for reflecting real-world complexities and choices within future equity assessments.

## 5 Conclusion

In this study, we integrate a top-down, long-term scenario analysis with a bottom-up, dynamic coal mine database for China to design and evaluate two coal mine phaseout strategies under carbon neutrality. This comprehensive approach enables a detailed examination of stranded asset costs and health benefits across Chinese provinces, shedding light on the complex trade-offs between efficiency and equity in pursuing carbon neutrality. We find that coal mine phaseouts generate health benefits worth 3478 to 3689 billion yuan, about 1% of the projected GDP for that year. However, the ERS strategy introduces serious regional disparities. Despite its intention to evenly distribute the economic impacts of mine phaseouts, the ERS disproportionately increases financial burdens on core coal-producing regions. By 2060, stranded asset costs in these regions are projected to rise to 1139.4 billion yuan under the ERS, which is 25.8% higher than the 905.8 billion yuan under the EPS. At the same time, the ERS reduces health benefits in these regions to 340.2 billion yuan, an 11.8% drop from the 389.7 billion yuan under the EPS. Our findings indicate that the ERS can unintentionally widen regional gaps and hinder the achievement of a balanced, just path to carbon neutrality.

## CRediT authorship contribution statement

**Bin Zhang:** Writing – review & editing, Writing – original draft, Supervision, Project administration, Formal analysis, Conceptualization. **Niu Niu:** Writing – original draft, Formal analysis, Data curation, Conceptualization. **Qingyao Xin:** Writing – original draft, Formal analysis, Data curation. **Yang Lu:** Writing – original draft, Formal analysis, Data curation. **Heran Zheng:** Writing – original draft, Supervision, Formal analysis. **Zhaohua Wang:** Writing – review & editing, Supervision, Project administration, Data curation, Conceptualization.

## Declaration of competing interest

The authors declare that they have no conflicts of interest in this work.

## Appendix Supplementary materials

Image, application 1
