# Supplementary material for: Evaluating the economic and social impact of coal mine phaseout in China
Source: Fundam Res. 2025 Sep 11;6(5):3330–9. doi: 10.1016/j.fmre.2025.09.001 (PMC13644501; doi:10.1016/j.fmre.2025.09.001)
Supplement: Supplementary file 1 [file mmc1.docx]

## Evaluating the Economic and Social Impact of Coal Mine Phaseout in China

## Note S1. Coal Mines Dataset of China

This study employs a comprehensive dataset comprising mine-source level data for coal mines operational within the Chinese territory as of April 2023. The foundational dataset was procured from the Global Energy Monitor (<https://globalenergymonitor.org/> ), a publicly accessible database. This initial dataset underwent rigorous modifications and updates, which were independently conducted using the annual reports of coal mining capacities issued by the National Energy Administration of China (<https://www.chinamine-safety.gov.cn/xw/zt/2018zt/mkscnlgl/index.shtml>), supplemented with data from additional resources such as Wikipedia. Following meticulous data consolidation and curation, the resultant database now includes 1,219 operational coal mines with an aggregated output of 3.76 gigatons in the year 2020. As reported by the “2020 Annual Report on Coal Industry Development [1] ” from the China Coal Industry Association, the national output for coal at the year’s end was 3.9 billion tones, indicating that our dataset captures over 96% of the total national coal extraction volume. The database provides granular details on each mine, encompassing Mine Type, Coal Type, Coal Grade, Annual Coal Output, Total Reserves, operational commencement date, affiliated province, and exact GPS coordinates. Figure S1 illustrates the geographical distribution of the mines, their annual production rates, and the projected remaining operational lifespan.

**Supplementary Fig. S1. Distribution of coal mines in China in 2020.**

Due to limitations in data collection, this map excludes coal mine data from Hong Kong, Macau, and Taiwan. Each circle on the map represents a coal mine, with the size of the circle indicative of the mine’s production capacity. The color of the circle denotes the remaining operational lifespan of the mine. The background color of the map reflects the Gross Domestic Product (GDP) of each province in the year 2020.

## Note S2. Global Change Assessment Model predictions for national coal mining output

The Global Change Assessment Model (GCAM) is a dynamic, recursive, and open-source integrated assessment model designed to analyze the intricate interactions among various sectors, including energy, land, water, and climate systems, and their connections to the Earth’s system (https://jgcri.github.io/gcam-doc/). The GCAM model divides the globe into 32 regions, with China being one of them. The model operates in five-year intervals from 2015 to 2100, continuously seeking to establish equilibrium prices that balance supply and demand across all sectors and regions. As a technology-intensive, bottom-up model, GCAM offers a comprehensive representation of the global energy system, encompassing a wide range of technologies and fuels, and covering the full spectrum of energy production, conversion, and consumption across multiple regions and sectors. It thoroughly considers both currently mainstream technologies and those under development. In GCAM, primary energy includes both fossil fuels like coal and renewable sources such as solar energy. For coal, the cost follows a cumulative supply curve, where extraction becomes progressively more difficult and costly. Different supply levels correspond to different extraction costs. Furthermore, as technology advances, the extraction costs for the same grade of supply may decrease.

GCAM determines the resource supply curves and extraction costs for each region based on the following equation (see Equation S1):

$P_{r,t,m}=\frac{C_{r,t,m}}{Tec_{t,m}}+Cenv_{r,t,m}$ S1

Where, $C_{r,t,m}$ represents the extraction cost of energy type $t$ in region $r$ for year $m$, $Cenv_{r,t,m}$ denotes additional costs incurred due to environmental policies, and $Tec_{t,m}$ is the rate of technological progress (see Equation S2):

$Tec_{t,m}=Tec_{t,m-1}\times\left(1+BT_{r,t}\right)^{STEP}$ S2

Where, $BT_{r,t}$represents the rate of technological progress for energy type $t$ in region$r$,$STEP$ refers to the computational step interval of GCAM, which is 5 years.

Different production activities require varying proportions of energy. In GCAM, the shares of energy consumption for different production activities and the technology shares under each type of energy are determined based on the Logit function (see Equation S3). The Logit function incorporates price factors into the model, thereby preventing the anti-market phenomenon of a single technology dominating the market.

$Share_{u}=\frac{Pr_{u}\times TC_{u}^{PI}}{\sum_{u} \left(Pr_{u}\times TC_{u}^{PI}\right)}$ S3

Where represents the sum of fuel and other costs for technology$u$. $PI$ is the price elasticity coefficient, which indicates the elasticity of a technology’s share in response to cost changes in another technology; $Pr$ is the base weight of technology $u$ which is exogenously adjusted. Finally, once the supply and demand for primary energy across all regions are balanced, the GCAM model calculates the prices corresponding to each type of primary energy.

Socioeconomic development, technological progress, and climate change are the three main factors influencing carbon emissions. In this study, we employ Shared Socioeconomic Pathway [2] (SSPs) to describe the socioeconomic development scenarios in China (including population and GDP) under different conditions. Since SSP scenarios do not include climate constraints, this paper follows Fuhrman et al.[3], to achieve net-zero emissions in China by 2060. Specific scenario settings are detailed in Table S1. Coal production and the coal price index under different scenarios are presented in Table S2.

**Table S1. Scenario development**

| Scenario name | Shared Socioeconomic Pathways | Emission constraint | The Net CO2 emissions in 2060 | Scenario description |
| --- | --- | --- | --- | --- |
| Carbon neutrality target | SSP1 | Net-zero CO2 emissions in China in 2060 | 300.84 MT CO2 | Sustainable economic development and accelerated technological transformation. Assuming a linear decline in China’s carbon emissions from 2020 to 2060, the nation is projected to achieve net-zero carbon dioxide emissions by 2060. |

**Table S2. The Coal Output and Coal Price Index**

|  | 2020 | 2025 | 2030 | 2035 | 2040 | 2045 | 2050 | 2055 | 2060 |
| --- | --- | --- | --- | --- | --- | --- | --- | --- | --- |
| Coal Output (Units: EJ) | 75.7 | 64.0 | 54.2 | 42.1 | 32.8 | 22.6 | 15.2 | 10.5 | 7.5 |
| Coal Price Index  (2020 = 100) | 1.00 | 0.96 | 1.22 | 1.34 | 1.47 | 1.59 | 1.72 | 1.87 | 2.00 |

**Supplementary Fig. S2. Coal mine phaseout pathway in China under carbon neutrality targets.**

Solid lines represent the retirement paths of coal mines in each province under the Efficiency Priority strategy, while dashed lines indicate the paths under the Equitable Reduction strategy.

## Note S3. Coal Mine phaseout Strategy

We develop a coal mine phaseout algorithm based on multi-criteria decision-making [4 - 6]. Initially, a set of evaluation criteria is selected to assess each coal mine, with particular emphasis on the age of the mines. We employ a normalization equation to assign scores to each coal mine (see Equation S4). Scores closer to 0 indicate that a mine is older and should be prioritized for phaseout; scores closer to 1 suggest that a mine is newer and its phaseout can be deferred.

 S4

Regarding the production capacity of coal mines, we referenced the classification standards for coal mine production capacities outlined in the Coal Mine Production Capacity Management Methods issued by the National Energy Administration of China [7] . The production capacity of underground coal mines is divided into four tiers, and that of open-pit coal mines is divided into two tiers. For specific classification standards and scoring details, please refer to Table S3.

**Table S3. The Coal Mine Type Scoring**

| Type | Underground  Coal Output < 3 (Annual, Mt) | Underground  3 < Coal Output < 6 (Annual, Mt) | Underground  6 < Coal Output < 10 (Annual, Mt) | Underground  Coal Output > 10 (Annual, Mt) | Surface Coal Output < 4 (Annual, Mt) | Surface Coal Output > 4 (Annual, Mt) |
| --- | --- | --- | --- | --- | --- | --- |
| Score | 1 | 2 | 3 | 4 | 1 | 2 |

For Coal Grade, we classify coal mines into Met, Thermal, and Thermal & Met categories. Following the policy preferences for different types of coal stipulated in the “14th Five-Year Plan for Energy Conservation and Emission Reduction Comprehensive Work Plan” issued by the State Council of China, we assign values to different Coal Grades [8]. For specific classification standards and scoring details, please refer to Table S4. Ultimately, the sum of scores across these three dimensions serves as the comprehensive score for each coal mine (see Equation S5). The phaseout order of all coal mines is ranked based on this comprehensive score, with mines scoring lower prioritized for phaseout. We define this strategy of prioritizing the phaseout of mines that are less efficient and more polluting as the **Efficiency Priority strategy**.

$Comprehensive\text{ }Score=S_{year}+S_{Type}+S_{Grade}$ S5

**Table S4. The Coal Mine Grade Scoring**

| Coal Grade | Thermal | Thermal & Met | Met |
| --- | --- | --- | --- |
| Score | 1 | 2 | 3 |

Due to China’s unique resource endowments, provinces rich in coal often experience slow economic development and have a high dependency on coal [9]. Imposing the burden of coal mine phaseout s solely on high-producing coal regions could severely impact local economic development. Given the lack of a unified definition of fairness, it is challenging to manage the burden of coal mine phaseout s. Under current circumstances, we introduce the concept of absolute equity into the coal mine phaseout strategy, meaning that regardless of the province where the mine is located, its operational age, coal type, or extraction method, each coal mine equally shares the burden of phaseout. According to the requirements of the China National Energy Administration, a coal mine is closed when it fails to meet the minimum operational capacity[10]. We define this approach of uniformly distributing the burden of capacity reduction across all coal mines as the **Equitable Reduction Strategy**.

## Note S4. Estimation of Air Pollutant Emissions

We utilize the 2020 Multi-resolution Emission Inventory for China (MEIC), developed by Tsinghua University, as the foundational emission data (http://meicmodel.org.cn/#firstPage). MEIC is a comprehensive three-dimensional matrix encompassing pollutants (SO_2_, NO_x_, CO, NMVOC, NH_3_, BC, OC, PM_2.5_, PM_10_, and CO_2_), emission timing, and locations. It is important to note that MEIC does not include emissions related to coal mining activities. We calculate the emissions associated with coal mining by applying emission factors specific to each coal mine along with their annual production (see Equation S6).

$E_{m,t}=P_{m}\times F_{m,t}$ S6

where $E_{i,t}$ denotes the pollutant $t$emissions of coal mine$m$, $P_{m}$represents the annual output of coal mine$m$, and$F_{m,t}$ signifies the corresponding emission factor for pollutant $t$of mine$m$. For the emission factors, we referred to the survey data and the research conducted by Zhang et al.[11], obtaining pollutant emission factors for coal mining in each province, detailed in Table S5. Future coal production by province is derived from the GCAM model and the coal mine phaseout strategies implemented.

**Table S5. Coal Mine Pollutant Emission Factors**

| Region | PM_2.5_ | NO_x_ | SO_2_ | CH_4_ | PM_10_ |
| --- | --- | --- | --- | --- | --- |
| Anhui | 11.3 | 13.9 | 20.0 | 23.2 | 23.2 |
| Fujian | 56.8 | 22.8 | 143.8 | 116.7 | 116.7 |
| Gansu | 8.8 | 12.5 | 52.0 | 18.2 | 18.2 |
| Guizhou | 21.9 | 22.1 | 156.4 | 45.1 | 45.1 |
| Henan | 6.0 | 12.5 | 29.4 | 12.2 | 12.2 |
| Heilongjiang | 26.7 | 36.0 | 58.1 | 54.9 | 54.9 |
| Jilin | 17.2 | 21.6 | 62.5 | 35.3 | 35.3 |
| Jiangsu | 5.9 | 25.6 | 45.8 | 12.0 | 12.0 |
| Jiangxi | 8.4 | 36.9 | 96.5 | 17.3 | 17.3 |
| Liaoning | 19.1 | 23.9 | 52.8 | 39.3 | 39.3 |
| Inner mongolia | 4.7 | 5.1 | 18.6 | 9.8 | 9.8 |
| Ningxia | 2.5 | 10.8 | 24.3 | 5.0 | 5.0 |
| Qinghai | 10.3 | 15.9 | 36.1 | 21.0 | 21.0 |
| Shandong | 4.9 | 10.8 | 49.7 | 10.2 | 10.2 |
| Shanxi | 16.3 | 15.2 | 36.3 | 33.5 | 33.5 |
| Shaanxi | 5.8 | 17.4 | 25.3 | 11.9 | 11.9 |
| Sichuan | 51.0 | 21.8 | 122.5 | 104.7 | 104.7 |
| Xinjiang | 205.2 | 8.4 | 20.0 | 421.2 | 421.2 |
| Yunnan | 13.2 | 28.8 | 43.0 | 27.0 | 27.0 |

## Note S5. WRF-CAMx Model configuration

This paper employs the Weather Research and Forecasting Model V4.0 (WRF) to simulate the physical processes of the atmosphere, including variables such as wind speed, temperature, humidity, precipitation, and radiation. Detailed parameters within the WRF model—such as horizontal resolution, simulation area, chemical mechanisms, vertical profiles, boundary and initial conditions, meteorological parameterization schemes, and selection of natural emission sources—are provided in Table S6. The Comprehensive Air Quality Model with Extensions V6.5 is used to integrate atmospheric chemistry and physical processes with meteorological backgrounds, simulating the spatiotemporal variation of pollutants from various sources and thus assessing their impact on air quality. Details on the horizontal and vertical resolutions, meteorological-chemical mechanisms, aerosol modules, and various emission sources in the WRF model are also listed in Table S6. The WRF model supplies the CAMx model with high-resolution meteorological field inputs, such as wind speed, temperature, humidity, and precipitation, ensuring that the transport, dispersion, and deposition of air pollutants are simulated against an accurate meteorological backdrop.

**Table S6. WRF-CAMx Model configuration**

| Model | Parameter | Configuration |
| --- | --- | --- |
| WRF | Horizontal Resolution | 36 km * 36km |
|  | Vertical Levels | 30 sigma levels from surface to tropopause |
|  | Base year | 2020 |
|  | Time Step | 21600 s |
|  | Shortwave radiation | New Goddard scheme |
|  | Longwave radiation | RRTM scheme |
|  | Surface layer | Pleim-Xiu scheme |
|  | Cloud microphysics | WSM6 scheme |
|  | Radiation time step | 30 min |
|  | Convection | Kain-Fritsch scheme |
|  | Meteorological grid layer | 34 layers |
|  | Boundary layer | YSU scheme |
|  | IC and BC | NCEP |
|  | Planetary boundary layer | ACM 2 scheme |
| CAMx | Horizontal Resolution | 36 km * 36km |
|  | Vertical Levels | 12 sigma levels from surface to tropopause |
|  | Base year | 2020 |
|  | IC and BC | WACCM |
|  | Anthropogenic | MEIC for China; MIX and CMIP6 for other Asian countries |
|  | Biogenic emissions | MEGANv2.1 |
|  | Open biomass burning | FINN |
|  | Gas-phase mechanism | CB05 |
|  | Aqueous-phase mechanism | RADM |
|  | Aerosol module | AERO6 |
|  | Cloud module | ACM_AE6 ACM cloud processor |
|  | Photolytic rate | In-line calculation |

## Note S6. The health benefit of coal mine phaseout

Currently, the exposure-response functions used for health impact assessments primarily include log-linear equations, the non-linear Integrated Exposure-Response (IER) function, and the Global Exposure Mortality Model (GEMM) [12,13]. GEMM, which has evolved from log-linear equations, is widely used at present and is designed to estimate mortality due to exposure to outdoor PM_2.5_. This function was developed based on data from 41 cohort studies on outdoor air pollution across 16 countries globally [13]. The relationship between PM_2.5_ concentration and mortality for specific cohorts is represented through a relative risk function, as shown in Equation S7.

$RR\left(C\right)=e^{\frac{\theta\times\ln\left(\frac{z}{\alpha}+1\right)}{1+e\left(-\frac{z-\mu}{\nu}\right)}},Z=\max\left(0,C-2.4\right)$ S7

Where,$C$ represents the ambient PM_2.5_ concentration,$\theta$,$\alpha$,$\mu$,$\nu$ are used to define the shape parameters for the RR within the GEMM for different age groups, $e$denotes the natural logarithm. In alignment with recent scholarly findings, the theoretical baseline for minimum risk exposure to PM_2.5_ employed in our study corresponds to 2.4 µg/m³ [14 - 16]. The estimated mortality rates for population groups within grid $i$ categorized by gender $g$ and age$a$ are computed as Equation S8:

$Mort_{i,j,g,k}=\sum_{d} \frac{RR\left(P\right)_{i,y,j,d}-1}{RR\left(P\right)_{i,y,j,d}}\times POP_{i,j,g}\times MB_{i,j,g,k}$ S8

Where $Mort_{i,j,g,k}$ represents the number of deaths in grid$i$ for age group$j$, gender$g$,and disease $k$; $POP_{i,j,g}$denotes the population count for age group $j$and gender$g$ in grid $i$; $MB_{i,j,g,k}$indicates the morbidity rate for disease $k$ in age group $j$and gender$g$ within grid $i$. According to existing research^17^, the historical and projected future baseline mortality rates for various diseases across different age groups are shown in Table S7.

**Table S7. The baseline Mortality Rates in 2020 and 2060**

| Cause | Year | Gender | 1 – 4 | 5 – 9 | 10 – 14 | 15 – 19 | 20 – 24 | 25 – 29 | 30 - 34 | 35 - 39 |
| --- | --- | --- | --- | --- | --- | --- | --- | --- | --- | --- |
| COPD | 2020 | female | 0.00000017 | 0.000000204 | 0.000000203 | 0.000000996 | 0.0000016 | 0.000002 | 0.0000039 | 0.0000072 |
| IHD | 2020 | female | 0 | 0 | 0 | 0.0000057 | 0.0000136 | 0.000016 | 0.0000288 | 0.0000476 |
| LC | 2020 | female | 0 | 0 | 0.000000341 | 0.00000167 | 0.00000351 | 0.00000629 | 0.0000144 | 0.0000317 |
| LRI | 2020 | female | 0.000151 | 0.00000734 | 0.00000369 | 0.00000318 | 0.00000381 | 0.00000352 | 0.00000375 | 0.00000521 |
| Stroke | 2020 | female | 0.0000025 | 0.00000174 | 0.00000188 | 0.00000683 | 0.0000115 | 0.0000146 | 0.0000251 | 0.0000494 |
| COPD | 2020 | male | 0.000000238 | 0.000000282 | 0.000000276 | 0.0000015 | 0.00000278 | 0.00000346 | 0.00000701 | 0.0000119 |
| IHD | 2020 | male | 0 | 0 | 0 | 0.0000131 | 0.0000345 | 0.0000538 | 0.000108 | 0.000208 |
| LC | 2020 | male | 0 | 0 | 0.000000403 | 0.00000253 | 0.00000584 | 0.0000116 | 0.0000269 | 0.0000564 |
| LRI | 2020 | male | 0.000176 | 0.00000883 | 0.0000046 | 0.00000605 | 0.00000783 | 0.00000665 | 0.00000932 | 0.000014 |
| Stroke | 2020 | male | 0.00000306 | 0.00000257 | 0.00000251 | 0.0000173 | 0.0000356 | 0.0000416 | 0.0000921 | 0.000173 |
| COPD | 2060 | female | 7.76E-08 | 8.76E-08 | 9.48E-08 | 0.000000481 | 0.000000803 | 0.00000095 | 0.00000252 | 0.00000442 |
| IHD | 2060 | female | 0 | 0 | 0 | 0.00000276 | 0.00000686 | 0.00000762 | 0.0000186 | 0.0000292 |
| LC | 2060 | female | 0 | 0 | 0.00000016 | 0.000000807 | 0.00000177 | 0.00000299 | 0.00000931 | 0.0000195 |
| LRI | 2060 | female | 0.0000692 | 0.00000315 | 0.00000171 | 0.00000154 | 0.00000192 | 0.00000168 | 0.00000242 | 0.00000319 |
| Stroke | 2060 | female | 0 | 0 | 0 | 0.000000177 | 0.000000452 | 0.000000522 | 0.0000012 | 0.00000215 |
| COPD | 2060 | male | 7.91E-08 | 9.89E-08 | 0.000000102 | 0.00000062 | 0.00000118 | 0.00000133 | 0.00000365 | 0.0000059 |
| IHD | 2060 | male | 0 | 0 | 0 | 0.00000545 | 0.0000146 | 0.0000208 | 0.0000564 | 0.000103 |
| LC | 2060 | male | 0 | 0 | 0.000000149 | 0.00000105 | 0.00000247 | 0.00000449 | 0.000014 | 0.0000279 |
| LRI | 2060 | male | 0.0000594 | 0.0000031 | 0.0000017 | 0.00000251 | 0.00000331 | 0.00000256 | 0.00000484 | 0.00000691 |
| Stroke | 2060 | male | 0 | 0 | 0 | 0.000000132 | 0.000000449 | 0.000000732 | 0.00000282 | 0.0000049 |

**Table S7. The baseline Mortality Rates in 2020 and 2060** **(Continued)**

| 40 - 44 | 45 - 49 | 50 - 54 | 55 - 59 | 60 - 64 | 65 – 69 | 70 – 74 | 75 – 80 | 80+ | Average Value |
| --- | --- | --- | --- | --- | --- | --- | --- | --- | --- |
| 0.0000154 | 0.0000282 | 0.0000628 | 0.000133 | 0.00032 | 0.000741 | 0.001984 | 0.004319 | 0.013822 | 0.001261275 |
| 0.0000891 | 0.00014 | 0.000257 | 0.000478 | 0.000935 | 0.001788 | 0.003749 | 0.007342 | 0.025825 | 0.002394988 |
| 0.0000769 | 0.000121 | 0.000227 | 0.000386 | 0.000665 | 0.001031 | 0.001574 | 0.002164 | 0.00295 | 0.000544283 |
| 0.00000722 | 0.00000895 | 0.000015 | 0.000025 | 0.0000456 | 0.0000922 | 0.000231 | 0.000547 | 0.002804 | 0.000232792 |
| 0.000106 | 0.000183 | 0.000382 | 0.000676 | 0.001263 | 0.002448 | 0.005073 | 0.009229 | 0.023256 | 0.002513503 |
| 0.0000294 | 0.0000569 | 0.000127 | 0.000278 | 0.000629 | 0.001384 | 0.003761 | 0.007864 | 0.022988 | 0.002184985 |
| 0.000349 | 0.000433 | 0.000739 | 0.001145 | 0.001824 | 0.002959 | 0.005746 | 0.010442 | 0.032384 | 0.003319906 |
| 0.00013 | 0.000231 | 0.00051 | 0.000957 | 0.001654 | 0.002586 | 0.004115 | 0.005355 | 0.007432 | 0.001357275 |
| 0.0000182 | 0.0000187 | 0.0000358 | 0.0000563 | 0.0000956 | 0.00018 | 0.000432 | 0.000956 | 0.004072 | 0.000358699 |
| 0.000315 | 0.000423 | 0.000804 | 0.001328 | 0.00233 | 0.004134 | 0.008505 | 0.014832 | 0.03465 | 0.003981691 |
| 0.00000807 | 0.0000161 | 0.0000403 | 0.0000862 | 0.000182 | 0.000554 | 0.001114 | 0.002568 | 0.012527 | 0.001006183 |
| 0.0000466 | 0.0000803 | 0.000165 | 0.00031 | 0.000533 | 0.001337 | 0.002106 | 0.004365 | 0.023406 | 0.001906702 |
| 0.0000403 | 0.0000692 | 0.000146 | 0.00025 | 0.000379 | 0.000771 | 0.000884 | 0.001287 | 0.002674 | 0.000384414 |
| 0.00000378 | 0.00000512 | 0.00000961 | 0.0000162 | 0.000026 | 0.000069 | 0.00013 | 0.000325 | 0.002541 | 0.000188854 |
| 0.00000401 | 0.0000111 | 0.0000279 | 0.0000589 | 0.000105 | 0.000265 | 0.000357 | 0.000553 | 0.001281 | 0.000156907 |
| 0.0000128 | 0.0000279 | 0.0000692 | 0.000159 | 0.000317 | 0.00089 | 0.001792 | 0.003975 | 0.018262 | 0.001501051 |
| 0.000153 | 0.000212 | 0.000404 | 0.000655 | 0.000918 | 0.001902 | 0.002738 | 0.005279 | 0.025726 | 0.002246309 |
| 0.0000566 | 0.000113 | 0.000279 | 0.000547 | 0.000833 | 0.001663 | 0.001941 | 0.002707 | 0.005904 | 0.000829039 |
| 0.00000796 | 0.00000916 | 0.0000195 | 0.0000322 | 0.0000481 | 0.000115 | 0.000206 | 0.000483 | 0.003234 | 0.000249368 |
| 0.00000815 | 0.0000194 | 0.0000384 | 0.000073 | 0.0000959 | 0.000208 | 0.000292 | 0.000475 | 0.001494 | 0.000159581 |

Further, we employed elasticity coefficients to estimate the calibrated values of the statistical life value for different provinces. The specific values are shown in Table S8.

**Table S8. Provincial VSL in 2020 and 2060 (Million Yuan, 2020 Price level)**

| Region | 2020 | 2060 |
| --- | --- | --- |
| Beijing | 5.46563682 | 14.5514059 |
| Tianjin | 5.657965996 | 19.31577805 |
| Hebei | 3.951895235 | 11.88445586 |
| Shanxi | 2.311238263 | 10.14583734 |
| Inner Mongolia | 2.855259416 | 12.97807391 |
| Liaoning | 3.175604611 | 10.52498788 |
| Jilin | 4.014617613 | 11.68280449 |
| Heilongjiang | 3.311287055 | 8.235657081 |
| Shanghai | 10.78405704 | 34.24964792 |
| Jiangsu | 7.032270419 | 20.38752352 |
| Zhejiang | 7.979781422 | 22.83710108 |
| Anhui | 2.312500834 | 8.131053436 |
| Fujian | 4.145401637 | 15.52016932 |
| Jiangxi | 2.149877338 | 8.129650384 |
| Shandong | 7.381902463 | 20.92178967 |
| Henan | 4.052107579 | 13.72683011 |
| Hubei | 2.332981065 | 9.025116872 |
| Hunan | 3.06382923 | 9.843736226 |
| Guangdong | 3.727933352 | 12.54558339 |
| Guangxi | 1.968438611 | 4.594286406 |
| Hainan | 2.910990583 | 8.960005699 |
| Chongqing | 2.975733803 | 10.29202369 |
| Sichuan | 2.637595299 | 9.844012653 |
| Guizhou | 1.330832493 | 2.805205351 |
| Yunnan | 1.953796733 | 5.816184797 |
| Tibet | 1.974233167 | 5.260182659 |
| Shaanxi | 2.839448153 | 9.095757995 |
| Gansu | 1.921160834 | 7.224964539 |
| Qinghai | 3.015389512 | 7.463755644 |
| Ningxia | 2.448211664 | 6.857817784 |
| Xinjiang | 2.078272744 | 6.106760486 |

## Note S7. Stranded Asset Costs

For coal mines, the financial losses due to asset stranding are a combination of stranded reserve costs and the associated economic losses from these stranded assets. The development of coal mines typically requires substantial upfront capital investments [18], including expenses related to exploration, infrastructure development, procurement of mining equipment, and ongoing operations and maintenance. However, due to stringent carbon emission regulations, coal that could have been extracted and brought to market remains unmined and stranded underground or in other storage locations. This results in a significant reduction, or even a complete cessation, of the expected cash flows. Moreover, fixed assets (such as mining land, equipment, and transportation infrastructure) may lose their economic value once resources are stranded, particularly high-cost infrastructure, which may face risks of impairment or underutilization.

The costs associated with resource stranding for coal mines are not proportionate to capital stranding costs. Estimating the cost of resource stranding necessitates the valuation of coal reserves. The market price of coal and the marginal cost of extraction are crucial factors in determining coal’s profitability; when the market price falls below the marginal cost, coal resources become economically unviable to mine, leading to stranding. To determine the market price of coal for 2020, we utilized data from the China Coal Market Network (https://www.cctd.com.cn/), and adjusted it according to the coal price index under the carbon-neutral scenario provided by the GCAM model, to estimate market prices for every five-year interval (with 2020 as the baseline). For the marginal cost of coal extraction, we relied on field surveys and the study “Research on Cost Curve of China’s Coal Industry” (https://pdf.dfcfw.com/) to obtain region-specific marginal extraction costs.

The evaluation of stranded resource costs typically employs a discounting method, which involves discounting future expected costs to their present value to quantify potential economic losses. The selection of the discount rate is critical as it directly impacts the present value estimation of future stranded costs. Existing studies indicate that more than 70% of professionals in the fossil fuel industry choose a discount rate in the range of 8%-12%, with over 60% opting for a 10% discount rate [19,20]. To further refine the choice of discount rate, we analyzed the financial statements of major coal mining companies to ascertain their asset discount rates (details provided in Table S9). On balance, a 10% discount rate for assets represents a reasonable and moderate choice.

**Table S9. Discount rate of fossil fuel enterprises**

| Enterprise / Research | Discount rate |
| --- | --- |
| China Shenhua Energy Company Limited [21] | 8.75% - 10.83% |
| China Coal Energy Group Co., Ltd [22] | 11.77% |
| Shanxi Coking Coal Group Company Limited [23] | 12.17% |
| China National Petroleum Corporation [24] | 6% - 12% |
| Saudi Arabian Oil Company [25] | 8% |
| Mercure et al., (2018) [26] | 10% |
| Kopacz et al., (2019) [20] | 10% |

In addition, the service life and actual operational duration of a coal mine determine the time span of stranded resources. If a coal mine is prematurely closed, more coal will become stranded. The service life of a coal mine determines how many years the enterprise can generate revenue from it. If the actual operational time of the coal mine is shorter than its designed service life, the unexploited coal resources will become stranded, leading to economic losses. The lifespan of a coal mine primarily depends on resource reserves, mining technology, and policy conditions. The design life of medium and large coal mines is generally long (typically over 50 years) [27]. Taking into account the impact of technological progress and coal capacity reduction, we set the lifespan of coal mines at 50 years. Consequently, we propose a method for calculating the stranded resource cost for coal mines (see Equation S9).

$FR_{m}=\sum_{y=0}^{Y} \frac{\left(C_{y}-MPC_{y}\right)\times TR_{m}\times O_{m,y}\times\frac{RY_{m}}{SC_{m}}}{\left(1+d\right)^{y}}$ S9

Where, $FR_{m}$denotes the cost associated with stranded reserves, $c_{y}$represents the market price of coal in $y$year, $MPC_{t}$ stands for the marginal cost of extraction in $y$year, $TR_{m}$indicates the proven reserves of coal mine $m$, and $O_{m,y}$signifies the proportion of unmined coal in year $y$relative to the proven reserves of coal mine $m$. When a mine reaches its service life, the coal within that mine will no longer be included in the calculation of stranded resources. The discount rate ($d$) is set at 10%.

For capital stranding costs, this study assumes that no new coal mines will be developed in the future and that none of the current coal mines will receive further investments. The unused portion of fixed assets results in financial losses. By matching the coal mines in the dataset with the total fixed asset value of the coal mining industry in each province, the fixed asset value of each coal mine was obtained. The capital stranding costs are calculated through discounting, specifically by discounting the present value of the coal mine’s fixed assets to a future year. The discount rate reflects the time value of money, and the same discount rate used for stranded resources is applied here.

**Supplementary Figure S3. Provincial GDP and per capita GDP of China in 2020, 2040, and 2060.**

**Supplementary Figure S4. Reductions in simulated annual PM_2.5_ concentrations from 2020 to 2060.**

The colors in the figure represent the reduction in PM_2.5_ emissions caused by coal mine phaseout s between 2020 and 2060 (Units: μg/m^3^). Lighter colors indicate a greater reduction in PM_2.5_ due to coal mine phaseout s in the region.

**Supplementary Figure S5. Avoided Mortality Due to Coal Mine phaseouts.**

The color gradient in the figure illustrates the estimated number of deaths averted as a result of coal mine phaseouts between 2020 and 2060 (measured in individuals). Regions shaded in blue represent areas with fewer avoided deaths, while regions shaded in red denote areas with a higher number of avoided deaths.

## Note S8. Sensitivity analysis

We conducted a systematic sensitivity analysis, varying key assumptions related to economic and environmental health assessments, to evaluate the robustness of our main findings. This analysis encompassed four critical parameters, detailed below:

（1） Discount rate. Informed by the observed range of discounting practices and the discussion in Table S9, we adopt a baseline discount rate of 10% and conduct sensitivity tests using a ±20% adjustment range, resulting in low (8%) and high (12%) scenarios.
（2） Future coal price. Future coal prices are subject to significant uncertainty due to global market dynamics, domestic energy policies, and fuel substitution patterns. Informed by prior modeling studies and price indices embedded within the GCAM, we construct high and low coal price scenarios by adjusting the baseline price by ±10%. These alternative trajectories are used to assess the sensitivity of stranded asset estimates to market price fluctuations.
（3） Value of Statistical Life. VSL reflects individuals’ willingness to pay for risk reductions. To account for uncertainty in this metric, we implement an alternative high-VSL scenario derived from established national and international estimates^28^, representing the upper bound of plausible valuations. This allows us to evaluate the extent to which our conclusions regarding net benefits are robust to variations in benefit monetization assumptions.

The sensitivity analysis reveals how variations in key parameters influence the estimated stranded asset costs and health benefits, providing insights into the robustness of our findings (Supplementary Figs. S6-S9). Variations in the discount rate show notable effects on Stranded Capital (Supplementary Figs. S6 and S7). Under the EPS, the estimated range for Stranded Capital is 596.14–872.52 billion yuan. A lower discount rate particularly increases estimated Stranded Capital in provinces such as Anhui and Jiangsu (by over 140%). Under the ERS, the Stranded Capital range is wider, estimated between 783.30 and 1775.33 billion yuan, with Qinghai and Guangxi showing the greatest sensitivity to discount rate changes in relative terms. For Stranded Reserves, the analysis yielded ranges of 596.14–872.52 billion yuan under EPS and 783.30–1775.33 billion yuan under ERS . At the provincial level, the impact of discount rate changes on stranded costs appears relatively less pronounced for core coal-producing regions compared to certain other provinces. As for the Changes in future coal prices exert a larger influence on outcomes under the ERS than under the EPS (Supplementary Fig. S8). Specifically, this analysis indicates that total Stranded Assets across the tested price scenarios range from 9.35–11.40 billion yuan under EPS and 0.55–0.74 billion yuan under ERS. Finally, Adjusting parameters for the VSL estimation results in different ranges for monetized national health benefits by 2060 (Supplementary Fig. S9). Under the EPS, the estimated range becomes 3688.85–5106.49 billion yuan, while under the ERS, the range is 3478.40–4809.31 billion yuan.

**Supplementary Fig. S6. Sensitivity analysis of stranded capital with respect to the discount rate**

**Supplementary Fig. S7. Sensitivity analysis of stranded reserves with respect to the discount rate**

**Supplementary Fig. S8. Sensitivity analysis of future coal price**

**Supplementary Fig. S9. Sensitivity analysis of VSL**

## Note S9. The glossary of key terms

| Term | Abbreviation | Explanation |
| --- | --- | --- |
| Efficiency Priority Strategy | EPS | A coal mine phase-out strategy prioritizing the sequential closure of mines based on performance scores, targeting the most inefficient and polluting mines first to meet reduction goals. |
| Equitable Reduction Strategy | ERS | A stylized coal mine phase-out strategy representing a burden-sharing approach; mandates closure of mines <0.3 Mt/yr, then allocates remaining production cuts proportionally based on capacity. |
| Global Change Assessment Model | GCAM | A global integrated assessment model simulating energy, economy, land use, and climate interactions; used here to develop China’s carbon neutrality coal production pathways. |
| Weather Research and Forecasting Model coupled with the Comprehensive Air Quality Model with Extensions | WRF-CAMX | A coupled atmospheric modeling system used to simulate regional air pollution (specifically PM2.5 concentration changes) resulting from different coal mine phase-out scenarios. |
| Global Exposure Mortality Model | GEMM | An exposure-response model used to estimate the relative risks of mortality from specific diseases (NCDs, LRI, COPD, LC, Stroke) associated with long-term exposure to ambient PM2.5. |
| Representative Concentration Pathways | RCP | Scenarios depicting different trajectories of greenhouse gas concentrations, used as input for climate modeling. |
| Shared Socioeconomic Pathways | SSP | Scenarios describing plausible future socio-economic development trends, used to inform projections. |
| Value of Statistical Life | VSL | An economic valuation representing society’s willingness to pay for reductions in mortality risk; used here to monetize the health benefits (avoided premature deaths) from PM2.5 reduction. |

**Reference**

[1] China National Coal Association, Annual Report on the Development of Coal Industry in 2020, 2021.

[2] K. Calvin, B. Bond-Lamberty, L. Clarke, et al., The SSP4: A World of Deepening Inequality, Glob. Environ. Change 42 (2017) 284–296.

[3] J. Fuhrman, A. F. Clarens, H. McJeon, et al., The Role of Negative Emissions in Meeting China’s 2060 Carbon Neutrality Goal, Oxford Open Climate Change 1 (2021) kgab004.

[4] R. T. Brown, W. Beata, Comprehensive Chemometrics, Elsevier, 2009.

[5] R. Y. Cui, N. Hultman, M. R. Edwards, et al., A Plant-by-Plant Strategy for High-Ambition Coal Power Phaseout in China, Nat. Commun. 12 (2021) 1468.

[6] B. Adem Esmail, D. Geneletti, Multi-Criteria Decision Analysis for Nature Conservation: A Review of 20 Years of Applications, Methods Ecol. Evol. 9 (2018) 42–53.

[7] National Energy Administration of China, Coal Mine Production Capacity Management Methods, 2006.

[8] The State Council of the People’ s Republic of China, 14th Five-Year Plan for Energy Conservation and Emission Reduction Comprehensive Work Plan, 2021.

[9] B. Lin, Y. Song, Coal Price Shocks and Economic Growth: A Province-Level Study of China, Energy Policy 193 (2024) 114297.

[10] National Development and Reform Commission, Work Plan for Classified Disposal of Coal Mines below 300,000 Tons/Year, 2019.

[11] Y. Zhang, X. Liu, L. Patouillard, et al., Remarkable Spatial Disparity of Life Cycle Inventory for Coal Production in China, Environ. Sci. Technol. 57 (2023) 15443–15453.

[12] R. T. Burnett, C. A. Pope, M. Ezzati, et al., An Integrated Risk Function for Estimating the Global Burden of Disease Attributable to Ambient Fine Particulate Matter Exposure, Environ. Health Perspect. 122 (2014) 397–403.

[13] R. Burnett, H. Chen, M. Szyszkowicz, et al., Global Estimates of Mortality Associated with Long-Term Exposure to Outdoor Fine Particulate Matter, Proc. Natl. Acad. Sci. U.S.A. 115 (2018) 9592–9597.

[14] C. J. L. Murray, A. Y. Aravkin, P. Zheng, et al., Global Burden of 87 Risk Factors in 204 countries and territories, 1990–2019: A Systematic Analysis for the Global Burden of Disease Study 2019, Lancet 396 (2020) 1223–1249.

[15] D. Tong, G. Geng, Q. Zhang, et al., Health Co-Benefits of Climate Change Mitigation Depend on Strategic Power Plant Retirements and Pollution Controls, Nat. Clim. Change 11 (2021) 1077–1083.

[16] Q. Zhao, X. Zhang, Y. Yang, et al., Revealing Inter-Regional Environmental Inequities Hidden in China’s Energy Transition, Environ. Sci. Technol. 57 (2023) 11852–11862.

[17] J. Cheng, D. Tong, Q. Zhang, et al., A Synergistic Approach to Air Pollution Control and Carbon Neutrality in China Can Avoid Millions of Premature Deaths Annually by 2060, One Earth 6 (2023) 978–989.

[18] C. Hauenstein, F. Holz, L. Rathje, et al., New Coal Mines in the Australian Galilee Basin Are Not Economically Viable and Are Prone to Become Stranded Assets, One Earth 6 (2023) 990–1004.

[19] T. A. Hansen, Stranded Assets and Reduced Profits: Analyzing the Economic Underpinnings of the Fossil Fuel Industry’s Resistance to Climate Stabilization, Renew. Sustainable Energy Rev. 158 (2022) 112144.

[20] M. Kopacz, J. Kulpa, D. Galica, et al., Economic Valuation of Coal Deposits – The Value of Geological Information in the Resource Recognition Process, Resour. Policy 63 (2019) 101450.

[21] China Shenhua Energy Co., Ltd., China Shenhua Energy Co., Ltd. Annual Report 2020, 2021.

[22] China Coal Energy Group Co., Ltd., Financial Statements and Audit Reports of China Coal Energy Group Co., Ltd., 2020.

[23] Shanxi Coking Coal Group Company Limited, Shanxi Coking Coal Group Company Limited Annual Report 2020, 2021.

[24] China National Petroleum Corporation, China National Petroleum Corporation Annual Report 2020, 2021.

[25] Saudi Aramco, Saudi Arabian Oil Company Consolidated Financial Statements for the Year Ended December 31, 2020, 2021.

[26] J.-F. Mercure, H. Pollitt, J. E. Viñuales, et al., Macroeconomic Impact of Stranded Fossil Fuel Assets, Nat. Clim. Change 8 (2018) 588–593.

[27] Ministry of Ecology and Environment of People’s Republic of China, The People’s Republic of China Second Biennial Update Report on Climate Change, 2019.

[28] Y. Jin, S. Zhang, An economic evaluation of the health effects of reducing fine particulate pollution in Chinese cities, Asian Dev. Rev. 35 (2018) 58–84.
